# Supplementary material for: Teneurins Are SPARCL1 Receptors
Source: bioRxiv. 2026 Jul 15:2026.07.13.738299. Preprint. [Version 1] doi: 10.64898/2026.07.13.738299 (PMC13405262; doi:10.64898/2026.07.13.738299)
Supplement: 1 [file NIHPP2026.07.13.738299v1-supplement-1.pdf]

bioRxiv preprint doi: https://doi.org/10.64898/2026.07.13.738299; this version posted July 15, 2026. The copyright holder for this preprint (which was not certified by peer review) is the author/funder, who has granted bioRxiv a license to display the preprint in perpetuity. It is made available under a CC-BY 4.0 International license.

SUPPLEMENTARY FIGURES & FIGURE LEGENDS

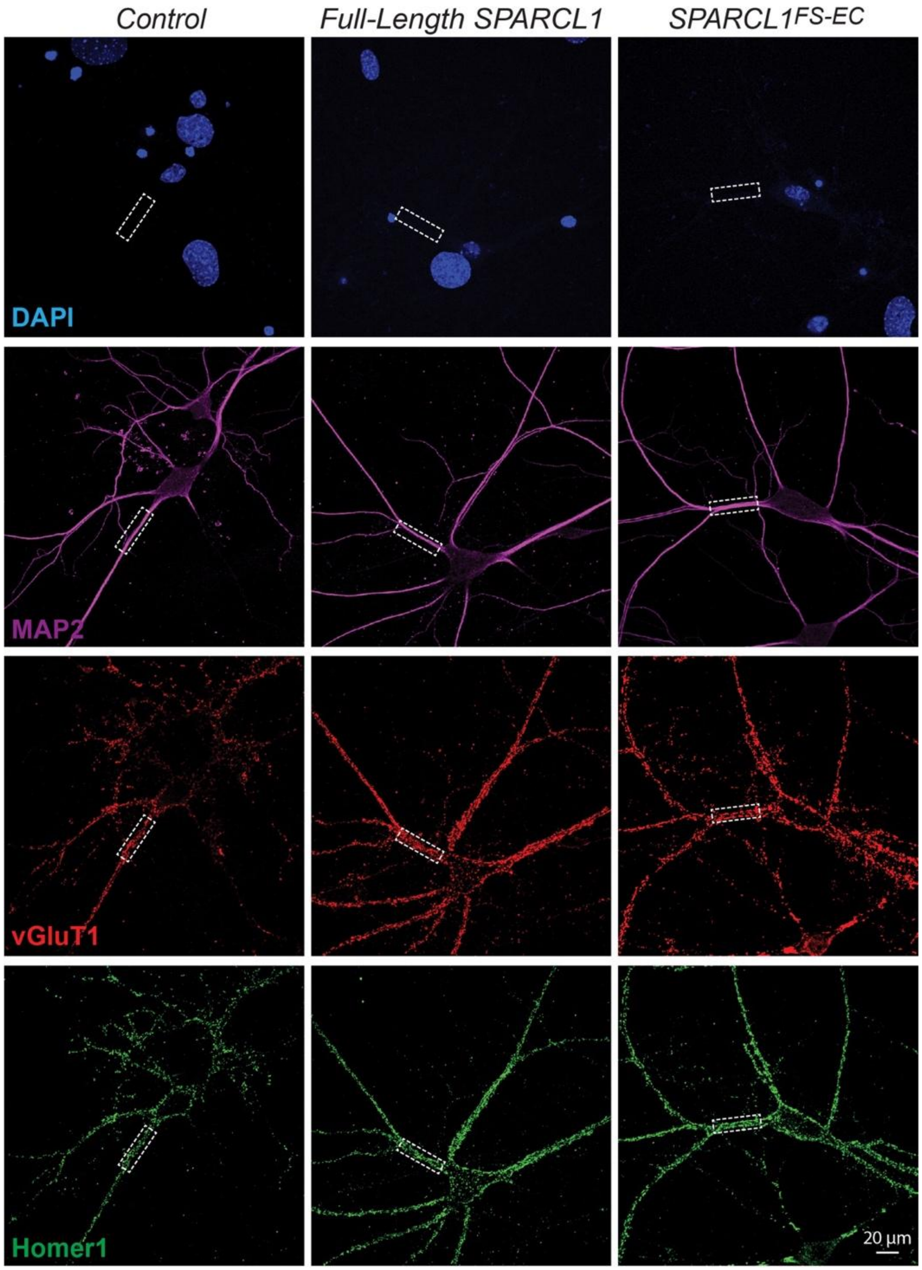

Figure S1 | **Single channel images for Figure 1**

Images depict the individual single channels for the data shown in Figure 1B. Neurons were stained for DAPI (blue), MAP2(Magenta), vGluT1 (red), and Homer1 (green). Left, with control medium; middle, with 50 nM full-length SPARCL1 protein; right, with 50 nM C-terminal SPARCL1^{FS-EC} protein.

bioRxiv preprint doi: https://doi.org/10.64898/2026.07.13.738299; this version posted July 15, 2026. The copyright holder for this preprint (which was not certified by peer review) is the author/funder, who has granted bioRxiv a license to display the preprint in perpetuity. It is made available under a CC-BY 4.0 International license.

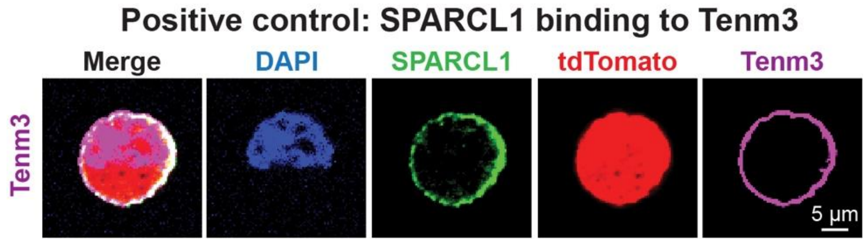

Figure S2 | **Additional positive control for Figure 3**

Representative images from experiments independently repeated 3 times showing that teneurin-3 (Tenm3) binds to SPARCL1.

bioRxiv preprint doi: https://doi.org/10.64898/2026.07.13.738299; this version posted July 15, 2026. The copyright holder for this preprint (which was not certified by peer review) is the author/funder, who has granted bioRxiv a license to display the preprint in perpetuity. It is made available under a CC-BY 4.0 International license.

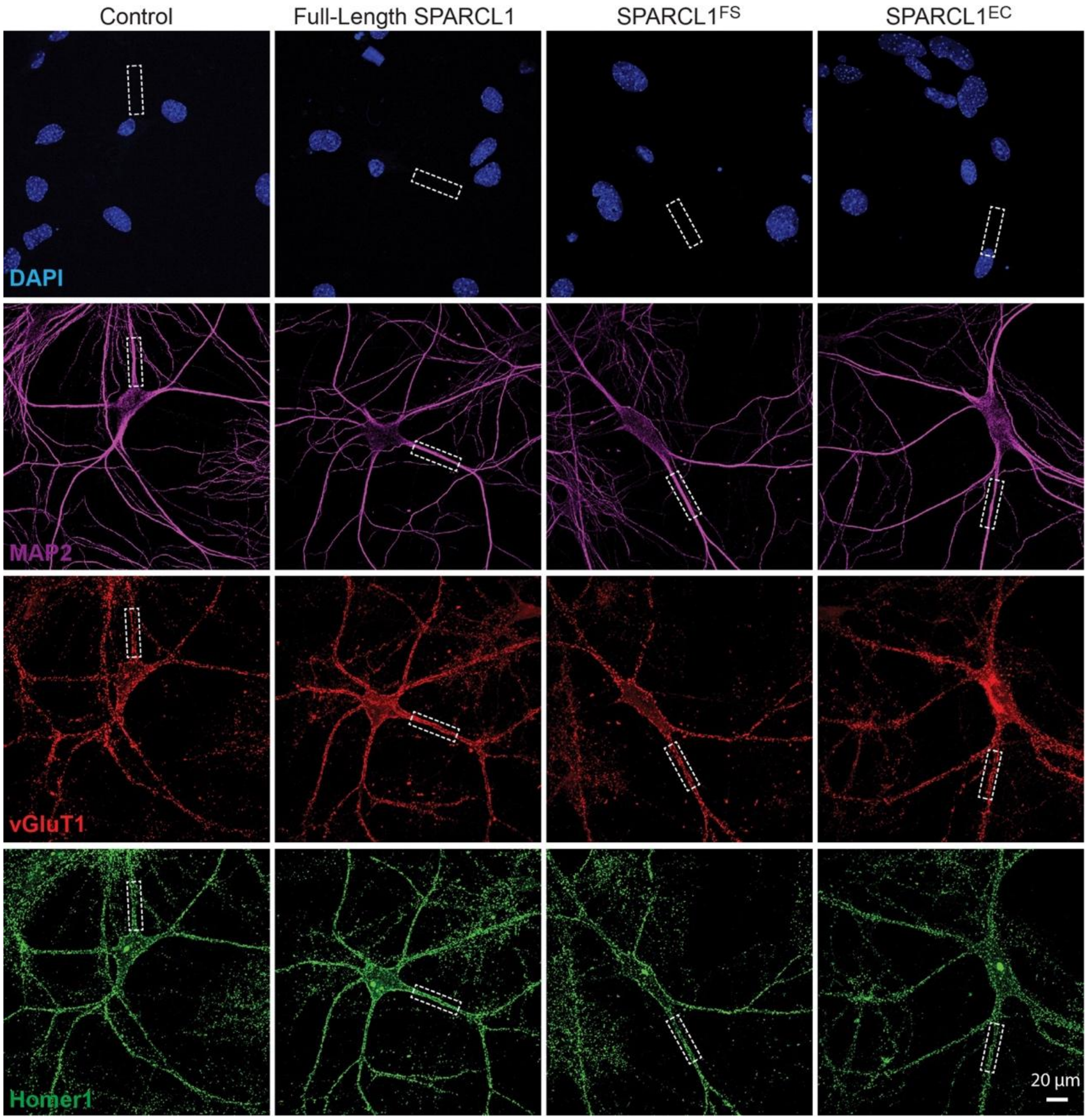

Figure S3 | **Single channel images for Figure 7**

Images depict the individual single channels for the data shown in Figure 7A. Neurons were stained for DAPI (blue), MAP2 (magenta), vGluT1 (red), and Homer1 (green). Left, with control medium; middle, with 50 nM full-length SPARCL1 protein; middle-left, with 50 nM full-length SPARCL1 protein; middle-right, with 50 nM SPARCL1^{FS}; right, with 50 nM SPARCL1^{EC} protein.
